# Efficient Sigma–Delta Sensor Array Beamforming

**DOI:** 10.3390/s23177577

**Published:** 2023-08-31

**Authors:** Sammy Johnatan Carbajal Ipenza, Bruno Sanches Masiero

**Affiliations:** 1 NXP Semiconductors N.V., 5656 Eindhoven, The Netherlands; 2 School of Electrical and Computer Engineering, Universidade Estadual de Campinas, Campinas 13083-852, Brazil

**Keywords:** MAXFLAT, PDM, sigma–delta, microphone, sensor array, decimation, beamforming

## Abstract

Nowadays, sensors with built-in sigma–delta modulators (ΣΔMs) are widely used in consumer, industrial, automotive, and medical applications, as they have become a cost-effective and convenient way to deliver data to digital processors. This is the case for micro-electro-mechanical system (MEMS), digital microphones that convert analog audio to a pulse-density modulated (PDM) bitstream. However, as the ΣΔMs output a PDM signal, sensors require either built-in or external high-order decimation filters to demodulate the PDM signal to a baseband multi-bit pulse-code modulated (PCM) signal. Because of this extra circuit requirement, the implementation of sensor array algorithms, such as beamforming in embedded systems (where the processing resources are critical) or in very large-scale integration (VLSI) circuits (where the power and area are crucial) becomes especially expensive as a large number of parallel decimation filters are required. This article proposes a novel architecture for beamforming algorithm implementation that fuses delay and decimation operations based on maximally flat (MAXFLAT) filters to make array processing more affordable. As proof of concept, we present an implementation example of a delay-and-sum (DAS) beamformer at given spatial and frequency requirements using this novel approach. Under these specifications, the proposed architecture requires 52% lower storage resources and 19% lower computational resources than the most efficient state-of-the-art architecture.

## 1. Introduction

In the last decades, sensor array processing has emerged as an active area of research in estimating space-time parameters. Array-processing applications are applied to solve many real-world problems. In telecommunications, for example, antenna arrays are steered in one user direction to reduce user interference. Radar and sonar use arrays of antennas and hydrophones, respectively, to calculate parameters like direction of arrival (DoA), velocity, and range. In medicine, sensor arrays are used for medical imaging, and planar biomagnetic sensor arrays are used in electrocardiograms to localize brain activity. In industry, sensor arrays are used in automatic monitoring and fault detection [1].

More recently, microphone array processing has emerged to increase the audio quality in consumer devices like mobile phones, speakerphones, and smart speakers, which are broadly used in conference rooms, desktop devices, and intelligent virtual assistants (IVA), in both consumer and industrial devices. Most frequently, the signals from several microphones are combined via a beamforming algorithm to enhance the sound coming from a desired direction while attenuating ambient noise and interference [1].

However, microphone array implementations are still expensive due to the complex characteristics of speech signals (non-static source, intermittent, and broadband) and the usual environmental conditions (reverberation and non-stationary additive noise). Adding an extra microphone in the design requires new routing, new placement conditions, and more processing resources, increasing the system cost and power consumption, a critical factor for internet of things (IoT) and mobile applications.

Digital MEMS microphones (introduced in 2006 [2]) have emerged as an alternative to overcome the size and cost limitations. As these microphones have an analog-to-digital converter (ADC) incorporated as a pre-amplifier [3], they have a single line PDM output; because of that, they are also known as PDM microphones (PDM-mics). A decimation filter (also known as a PDM-to-PCM converter) demodulates this PDM bitstream output to a PCM signal. Unfortunately, implementing this decimation filter is still not cheap, as its cost (measured in die area and power) increases with the quality of the desired audio signal. Take, for example, the case of a microphone array using these PDM-mics. This architecture requires a decimation filter for each microphone input so that the implementation cost and power consumption will increase proportionally with the number of microphones, being even more expensive for practical applications.

This paper proposes a novel and economical method to implement beamforming algorithms with arrays of MEMS digital microphones. We apply the new architecture to a DAS beamformer as a proof of concept, but it can also be used with other beamforming strategies. This method merges a conventional beamformer’s filtering and delays operations into a single structure dubbed as *delayed decimation filter*. We propose a *J*-stage decimation filter whose penultimate stage (J−1) is a Samadi filter, and its last stage (*J*) is an equiripple filter. The Samadi filter controls the overall filter delay by adjusting a single parameter, and the last equiripple stage compensates for the magnitude and phase distortion caused by the Samadi filter under a specific limit.

In the end, the proposed delayed decimation filter is an “all-in-one” filter that performs the same filtering and downsampling operations as any state-of-the-art decimation filter, has the capability of altering its group delay without any change in its structure or additional delay chain, and provides storage and computational resources savings in comparison to state-of-the-art architectures.

To explain the working principle of the proposal, we first recapitulate the implementation of a DAS beamformer in Section 2. We then present a novel beamformer based on delayed decimation filters in Section 3, where we introduce multirate and decimation filters, as well as how a Samadi filter can be used with these structures. To conclude, as a proof of concept, we present in Section 4 an implementation of this novel architecture, and in Section 5, we compare it to state-of-the-art DAS beamformer architectures.

## 2. DAS Beamformer

The DAS beamformer is the oldest and simplest array signal processing algorithm [1]. The underlying idea is to delay each microphone input by an appropriate time delay and then add all delayed microphone signals together. In this sense, the audio signal arriving from a particular direction at the array is reinforced in relation to signals coming from different directions and incoherent noise.

The traditional or discrete-time DAS beamformer (In the literature, the traditional DAS does not have the weights $w_{m}$ in its temporal representation because these weights only show up if you use a “weighted DAS” or a frequency representation; however, in this work the “weighted DAS” is referred to as the traditional DAS, as $w_{m}$ can implement the averaging process.) is the result of

(1) $$z\left[k\right]=\sum_{m=0}^{M-1}w_{m}y_{m}\left[k-k_{m}\right]\;,$$

where $y_{m}$ is the *m*th microphone’s output in PCM representation and $k_{m}$ is the integer delay associated with the *m*th microphone, such that

(2) $$k_{m}=[\Delta_{m}/T]=[\Delta_{m}f_{o}],$$

where $\Delta_{m}$ is the required delay in the *m*th microphone, [*x*] means the nearest integer to *x*, and $f_{o}$ and *T* are the sampling rate and period in $y_{m}$, respectively.

**Figure 1:**
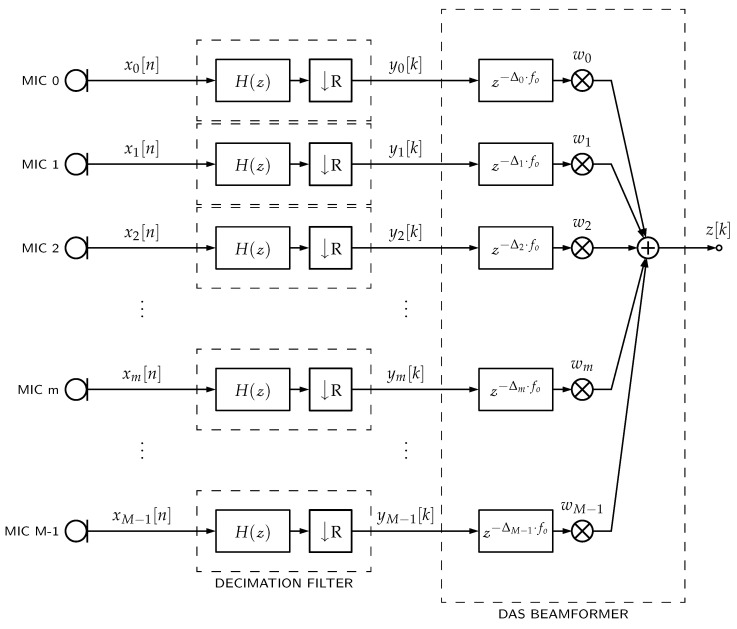
PDM microphones’ DAS beamformers. Each PDM-mic requires a decimation filter with H(z) frequency response and *R* downsampling. Then, each filter output $y_{m}\left[k\right]$ is delayed by a $\Delta_{m}$ factor. Finally, all delayed signals are weighted (factor $w_{m}$) and summed together.

In case of PDM-mics, Equation (1) can be represented as shown in Figure 1, such that $y_{m}$ is the decimation filter’s output and $x_{m}$ is the PDM bitstream incoming from the respective *m*th PDM-mic.

**Figure 2:**
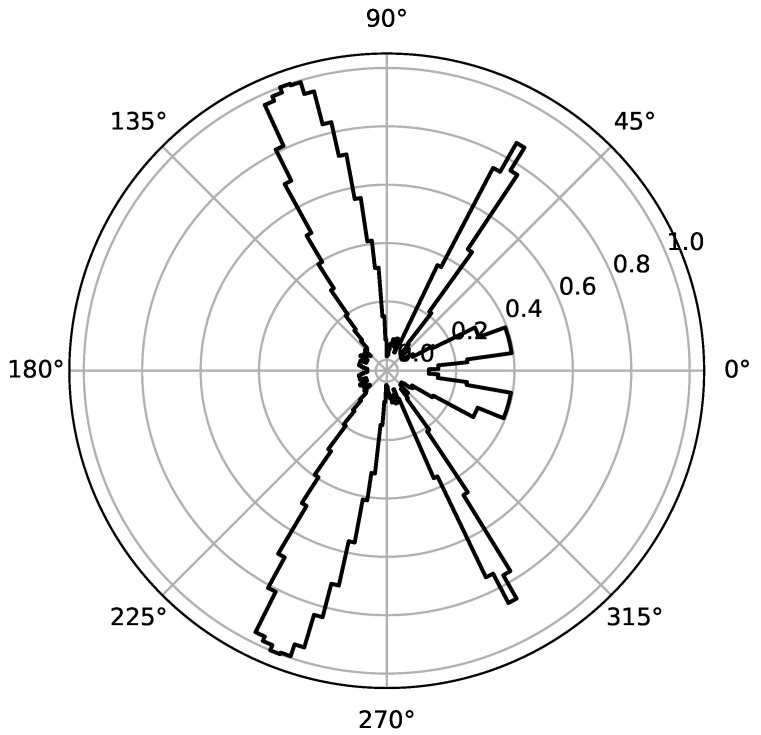
Normalized power (polar) of a uniform linear array of an M=40 microphones DAS beamformer. Three audio sources of 1 kHz, 3 kHz, and 5 kHz are located at 20, 60, and 110 degrees, respectively, i.e., the three with equal strength. The beamformer is placed on the X-axis. Therefore, its directivity pattern is symmetric about this axis.

Due to the integer nature of *k*, the DAS beamformer does not allow one to form sums that involve noninteger multiples of *T*. Consequently, beams cannot be steered in arbitrary directions, resulting in a directivity pattern with a stepped response due to the integer nature of the delay elements, which limits the beamformer resolution (as exemplified in Figure 2).

Also, if one assumes uncorrelated noise at the locations of the sensors and that the beamformer’s delays are appropriately matched to the wave’s DoA, it can be proven [4] that the beamformer gain (*G*) depends only on the weights $w_{m}$ and the number of microphones:

(3) $$G=\frac{{\left(\sum_{m=0}^{M-1}w_{m}\right)}^{2}}{\sum_{m=0}^{M-1}w_{m}^{2}}\;,$$

so that, for the beamformer in Figure 2, with M=40 and $w_{m}=1$, the white noise gain will be G=40 or 32 dB. Furthermore, the dynamic range depends only on the number of elements in the array. The array used for the current example provides a dynamic range of 13 dB.

## 3. Beamformer Based on Delayed Decimation Filter

Figure 1 describes a typical architecture for implementing DAS beamformers with PDM-mics. For each PDM-mic, there is an associated decimation filter to convert the PDM bitstream into a PCM bitstream and a delay line to steer the beamformer. To devise a more economical implementation of this architecture, we propose to merge the decimation filtering and the delaying operations into a single structure. To explain how a Samadi filter can be used for this purpose, we first review the concept of multirate and decimation filters, present the Samadi filter structure, show how it can be used as a multirate filter, and finally propose a new beamforming architecture based on this multirate filter (delayed decimation filter).

### 3.1. Multirate and Decimation Filters

**Figure 3:**
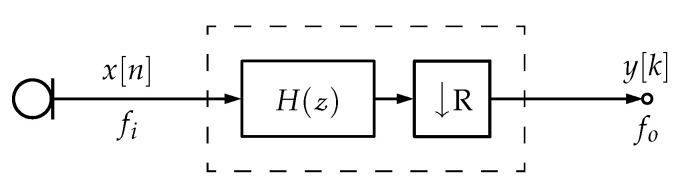
Generic decimation filter structure. In order to avoid aliasing, the input data x[n] at $f_{i}$ sampling rate is low-pass filtered and then downsampled by *R*. If correctly filtered, the output data y[n] at $f_{o}$ sampling rate contain the same information as x[n] decimated by *R*.

Multirate filters are digital filters whose different parts operate at different rates. The most obvious application of such a filter is when the input and output sample rates must differ (decimation or interpolation). A decimation filter is a class of multirate filters [5] that decreases a signal sampling rate by an integer or fractional factor. Figure 3 shows a generic decimation filter structure, where the input signal at $f_{i}$ sampling rate passes through a low-pass filter (LPF) with impulse response H(z), and then it is downsampled by a factor *R* to an output sampling rate $f_{o}=f_{i}/R$. In the case of a PDM-mic, usually, x[n] has a one-bit width only while y[k] is a multi-bit output.

**Figure 4:**
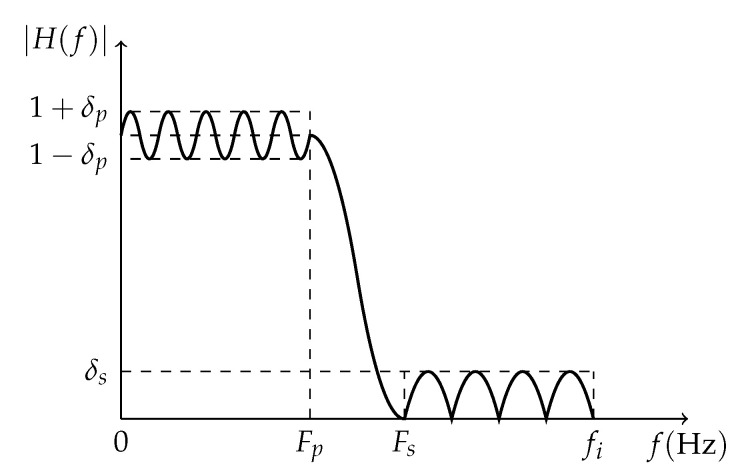
Low-pass filter design parameters. The passband and stopband regions are defined by $F_{p}$ and $F_{s}$, respectively, and their respectives ripples are defined by $\delta_{p}$ and $\delta_{s}$. The whole filter frequency response is constrained to the input sampling rate ($f_{i}$).

For a given application, there are many design parameters to be taken into account for the LPF design, such as filter passband frequency $F_{p}$, stopband frequency $F_{s}$, passband ripple $\delta_{p}$, and stopband ripple $\delta_{s}$, as exemplified in Figure 4. Those LPF design parameters are related as follows:

(4a) $$\begin{array}{cc} U_{p} & =\{f:f\in \left[0,F_{p}\right]\} \end{array}$$

(4b) $$\begin{array}{cc} U_{s} & =\{f:f\in \left[F_{s},f_{i}\right]\} \end{array}$$

(4c) $$\begin{array}{cc} \delta_{p} & =\max\left(||H(e^{2\pi if/f_{i}})|-1|\right)\;\forall f\in U_{p}, \end{array}$$

(4d) $$\begin{array}{cc} \delta_{s} & =\max\left(|H(e^{2\pi if/f_{i}})|\right)\;\forall f\in U_{s}, \end{array}$$

where $U_{p}$ and $U_{s}$ are the passband and stopband frequency ranges, respectively. Also, the angular passband and stopband frequencies can be expressed as

(5a) $$\begin{array}{cc} wp & =\frac{2\pi F_{p}}{f_{i}}, \end{array}$$

(5b) $$\begin{array}{cc} ws & =\frac{2\pi F_{s}}{f_{i}}, \end{array}$$

and $U_{p}$ and $U_{s}$ intervals can be scaled to angular frequency domain as

(6a) $$\begin{array}{cc} Vp & =\frac{2\pi U_{p}}{f_{i}}, \end{array}$$

(6b) $$\begin{array}{cc} Vs & =\frac{2\pi U_{s}}{f_{i}}. \end{array}$$

In the case of audio sensors such as MEMS microphones, a decimation filter is required to convert the oversampled output from the internal ADC to a standard audio PCM output. Baseband signal quality parameters such as linearity, signal-to-noise ratio (SNR), total harmonic distortion (THD), and total harmonic distortion plus noise (THD+N) can be worsened at the filter output if the LPF is not properly designed [6]. Also, the LPF structure should be carefully chosen to obtain a proper phase response. A Finite Impulse Response (FIR) structure, for example, can be used if a linear phase is required; otherwise, Infinite Impulse Response (IIR) filters are preferred, as, usually, IIR filters are smaller than their equivalent FIR implementations. Moreover, some applications tolerate some degree of non-linearity in phase; in this case, quasi-linear filters, a mixture of FIR and IIR filters, can be used.

### 3.2. Universal Maximally Flat Samadi Filter

As derived in [7], the transfer function in Samadi filters is defined by

(7) $$H_{N,K,d}\left(z\right)=\sum_{j=0}^{N-K}c_{j}{\left(\frac{1-z^{-1}}{2}\right)}^{j}{\left(\frac{1+z^{-1}}{2}\right)}^{N-j},$$

where

(8) cj=∑i=0j(−1)j−iN2−diN2+dj−i,

*K* is the number of zeros at z=−1, *N* is the filter order, and the delay parameter *d* is a real number defined as

(9) $$d=\alpha -\frac{N}{2}.$$

For a given group delay α, such that 0≤α≤N, from (9), one can verify that

(10) $$-\frac{N}{2}\leq d\leq \frac{N}{2}$$

or

(11) $$\left|d\right|\leq d_{\max}\;,$$

where $d_{\max}=N/2$ is the maximum allowed delay parameter and the binomial coefficients in (8) are defined as

(12) rs=∏q=0s−1r−qq+1,s≥11,s=00.s<0

This filter becomes a maximally flat (MAXFLAT) linear phase FIR when d=0. As shown in [8,9], the angular passband frequency (*w_p_*) of these linear phase filters is related with *N* as

(13) $$L≃\left\lceil Nw_{p}/\pi +0.5\right\rceil$$

where *L* is defined for convenience as

(14) L=N−K.

**Figure 5:**
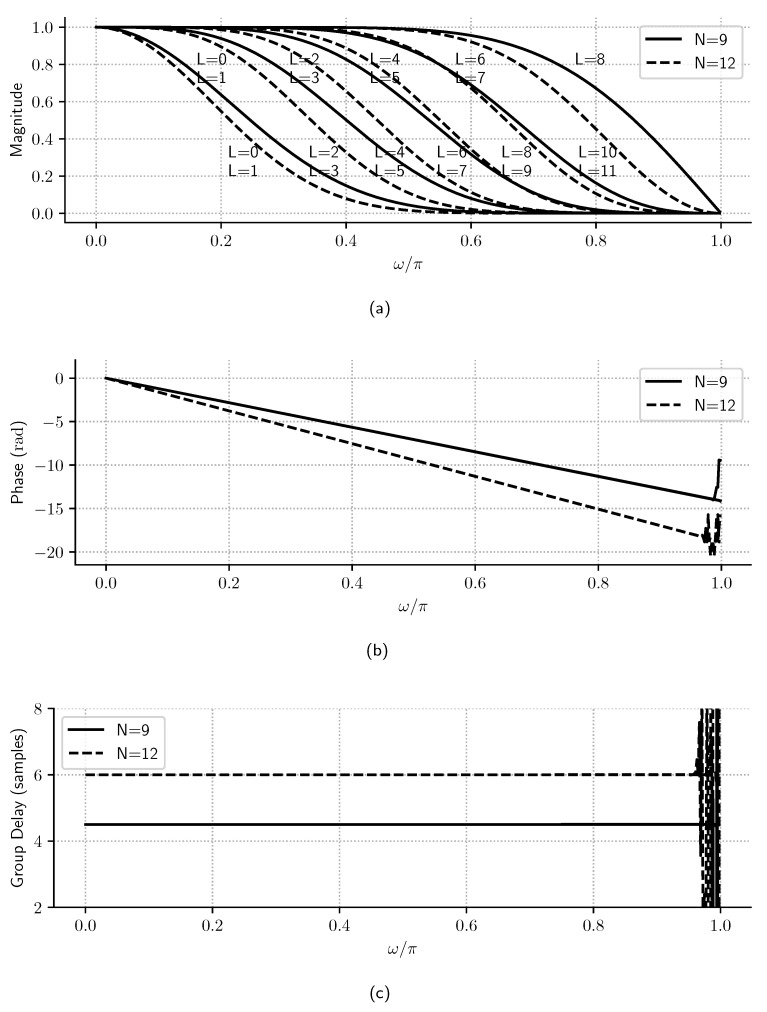
Normalized frequency spectra of linear-phase Samadi filters (d=0) with N=9 and N=12: (**a**) magnitude, (**b**) phase, and (**c**) group delay. It is observed that, in d=0 case, *w_p_* changes linearly with *L*, that the phase is linear for both *N* values and that the group delay is proportional to *N*.

The cutoff frequency of these linear phase filters increases almost linearly with *L*, as shown in Figure 5 for different values of *N*. Also, as demonstrated in [7] and shown in Figure 5a, for linear phase filters (d=0), the coefficient of (7) is

(15) $$c_{j}{|}_{d=0}=0,\;j\;\mathrm{odd}.$$

Then, the magnitude frequency spectra of L=2j and L=2j+1 are the same for j∈{0,…,⌊N/2−1⌋}. Figure 5 also shows that the filter has a linear phase and that the group delay for d=0 is α=N/2, as expected by (9).

**Figure 6:**
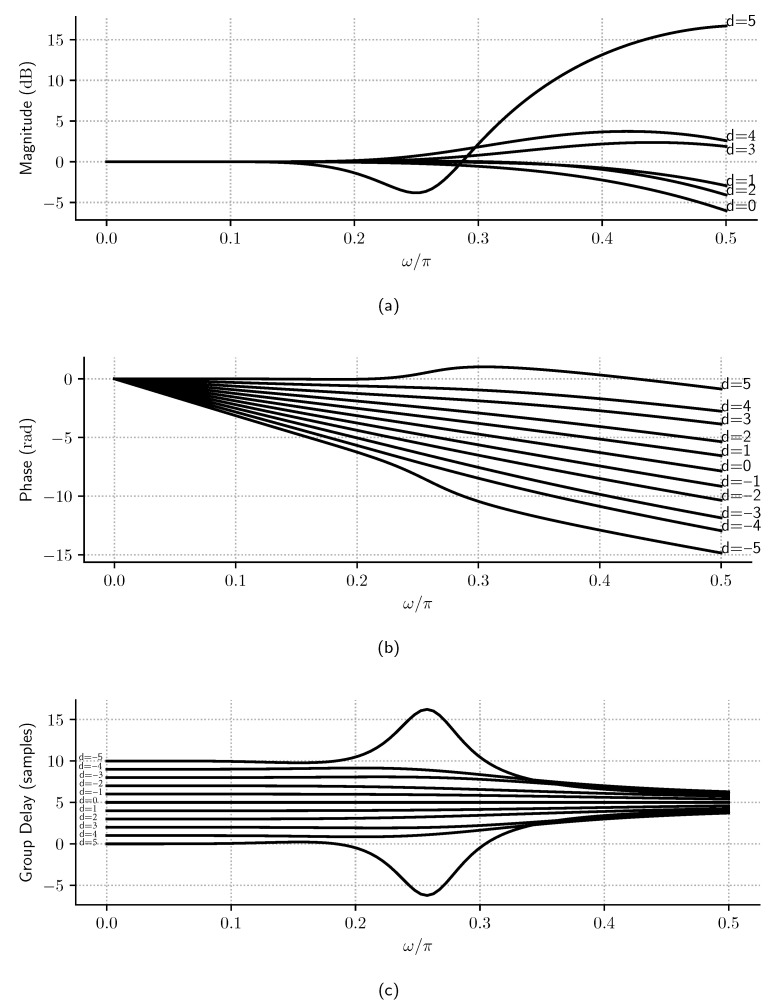
Normalized frequency spectra of Samadi filters with N=10 and d∈{−5,…,5}: (**a**) magnitude, (**b**) phase, and (**c**) group delay. It is observed that, approximately until ω/π<0.15, the magnitude is flat, the phase is linear, and the group delay is proportional to *d*. For ω/π≥0.15, the frequency response is nonlinear in magnitude, phase, and group delay.

On the other hand, when d≠0, the Samadi filter becomes a MAXFLAT nonlinear phase filter. The most interesting characteristic of this filter class is the ability to modify its group delay with the filter delay parameter (*d*), as given by (9). Figure 6 shows how the flatness of the magnitude and phase of the filter’s frequency response is affected when *d* increases—we see that passband $\delta_{p}$’s ripples worsen as *d* increases. However, it is also shown that the phase is still linear inside the passband region for ω<0.15π and that the decimation filter continues under the same specification for all values |d|≤5. This suggests that this filter can be used as an intermediary stage in a multirate filter chain to adjust the overall filter delay (Δ) and perform low-pass filtering at the same time, as discussed in the following sections.

Finally, we propose Algorithm 1 to calculate the minimum *K* and *N* Samadi filter values for a given *d*, matching a given filter specification with the following parameters: *V_p_*, *V_s_*, $\delta_{p}$, and $\delta_{s}$. In lines 2–4, the algorithm initializes *w_p_*, *L*, and *N* values to the minimum possible ones. Then, in line 5, it starts to iterate to calculate the minimum *K* and *N* values. In line 6, *K* is updated. In lines 7–8, $\delta_{p}$ and $\delta_{s}$ are calculated from the filter frequency response for *V_p_* and *V_s_* ranges, respectively, and for the current *K* and *N* values. If $\delta_{p}$ and $\delta_{s}$ meet the specification, it returns the parameter values in line 10. Else, in lines 12–26, it increases the *N* or *L* value, depending on the *d* weight or if the filter parameters are inside ranges defined in (13)–(15).
**Algorithm 1** Samadi Filter minimum *N* and *K* calculation algorithm 1:**procedure** SamadiMinN($d,\delta_{p},\delta_{s},V_{p},V_{s}$) 2:    L←0 3:    N←2⌈d⌉ 4:    $w_{p}\leftarrow \max(V_{p})$ 5:    **loop** 6:        K=N−L 7:        $\delta_{p}^{\prime }\leftarrow \max(|H_{N,K,d}\left(e^{i\omega }\right)-1\left|\right)\;\forall \omega \in V_{p}$ 8:        $\delta_{s}^{\prime }\leftarrow \max(|H_{N,K,d}\left(e^{i\omega }\right)\left|\right)\;\forall \omega \in V_{s}$ 9:        **if** $\delta_{p}^{\prime }\leq \delta_{p}$ **and** $\delta_{s}^{\prime }\leq \delta_{s}$ **then**10:           **return** N,K11:        **else**12:           **if** *d* = 0 **then**▹ Linear-phase filter13:               **if** $L>\lceil Nw_{p}/\pi +0.5\rceil$ **then**14:                   L←015:                   N←N+116:               **else**17:                   L←L+218:               **end if**19:           **else**▹ Nonlinear-phase filter20:               **if** $\delta_{s}^{\prime }\geq 1$ **or** L≥N **then**21:                   L←022:                   N←N+123:               **else**24:                   L←L+125:               **end if**26:           **end if**27:        **end if**28:    **end loop**29:**end procedure**

**Figure 7:**
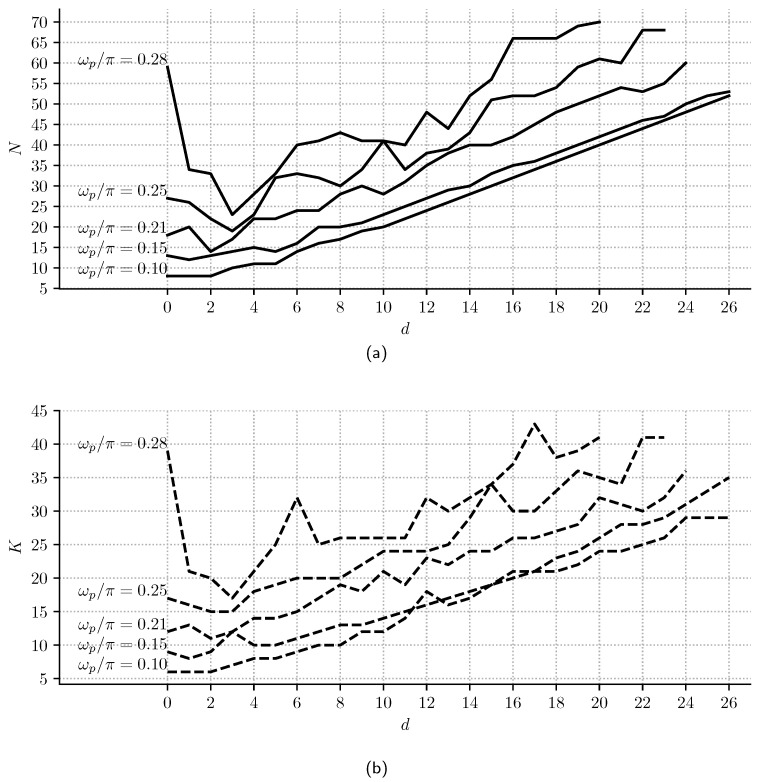
Minimum (**a**) *N* and (**b**) *K* values calculated using Algorithm 1 for $\delta_{s}=-80$ dB and different values of *d* and *w_p_*. It is observed that *w_p_* and *d* have a negative correlation for a given *N* value i.e., when *w_p_* increases, *d* decreases.

Figure 7 shows minimum *N* and *K* values, calculated using Algorithm 1 for d∈{0,…,26} and different values of *w_p_*. It is shown that the minimum *N*, required for any *d*, decreases with *w_p_* increments, and it is almost three times *d* when $w_{p}/\pi =0.28$.

Also, it is essential to remark that, if the Samadi filter is designed for $d_{\max}$, the decimation filter continues under the same specification for values $\left|d\right|\leq d_{\max}$. This effect can be observed in Figure 6a, where $\delta_{p}$ decreases for lower values of *d*, and, in Figure 7, where, for d≥3, if *N* is kept constant and *d* is decreased, *w_p_* tends to increase so that the flatness is improved.

### 3.3. Delayed Decimation Filter

**Figure 8:**
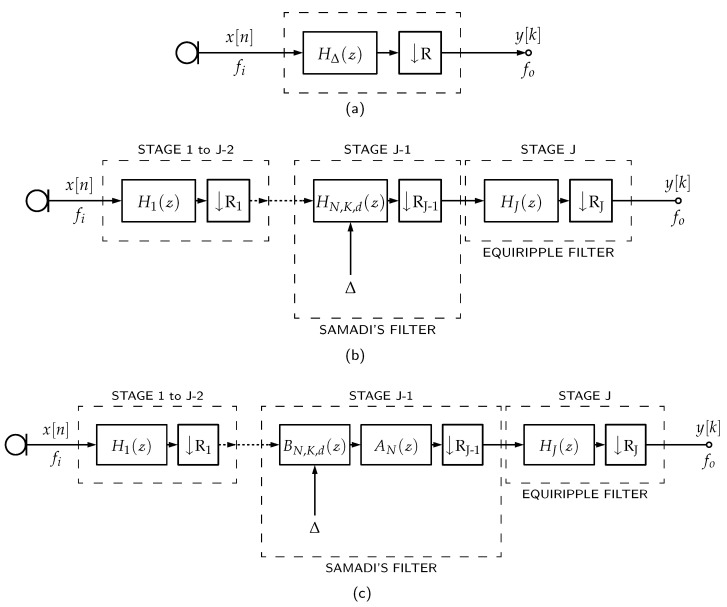
(**a**) Delayed decimation filter, (**b**) its version as a multi-stage decimation filter with the J−1 stage being a Samadi filter, and (**c**) its version with Samadi filter decomposed into its binomial components. Samadi filter stage is meant to control the overall filter delay (Δ) and the equiripple filter to compensate the non-linear response of the Samadi filter in its non-flat band. The optional Stages 1 to J−2 are meant to compensate and downsample the overall frequency response.

Because of its configurable group delay property, a single Samadi filter could be used as the LPF of a multirate filter with adjustable overall filter delay, as shown in Figure 8a—this structure is dubbed in this paper as delayed decimation filter. However, as a Samadi filter does not have the flexibility to be designed for specific $F_{p}$ and $F_{s}$ values without changing other filtering parameters, its frequency response needs to be compensated to keep the overall decimation filter’s parameters under specification for different delay values (*d*). For this reason, we propose a *J*-stages decimation filter architecture whose penultimate stage (J−1) is a Samadi filter and its last stage (*J*) is an equiripple filter, as shown in Figure 8b. The Samadi filter can then be decomposed iton its binomial components, as shown in Figure 8c.

The Samadi filter controls the overall filter delay (Δ) by setting its respective *d* parameter, and the last equiripple stage compensates for the magnitude and phase distortion caused by the Samadi filter under a specified limit. Also, as this is a multi-stage filter, other filtering stages (1 to J−2) can be optionally added to help with decimation and filtering.

The overall filter delay Δ depends on the *d*, $R_{J-1}$, and $R_{J}$ parameters in such a way:

(16) $$\Delta =\frac{d}{R_{J}R_{J-1}f_{o}}.$$

If we replace (16) in (11), it is observed that the maximum required delay ($\Delta_{\max}$) is limited by the $d_{\max}$ parameter as follows:

(17) $$\left|\Delta \right|\leq \frac{d_{\max}}{R_{J}R_{J-1}f_{o}}\;.$$

Therefore, since $d_{\max}=\Delta_{\max}R_{J}R_{J-1}f_{o}$, the minimum *K* and *N* parameters can be calculated using Algorithm 1 for $d=d_{\max}$ and the desired filter specification parameters: $\delta_{p}=\delta_{p}^{j},\delta_{s}=\delta_{s}^{j},V_{p}=V_{p}^{j}$, and $V_{s}=V_{s}^{j}$ for j=J−1.

### 3.4. Optimized Beamformer Structure

Since the Samadi filter is a binomial filter sequence (as first proposed by Haddad in [10]), (7) can be rearranged to allow the filter to be expressed as

(18) $$H_{N,K,d}\left(z\right)={\left(\frac{1+z^{-1}}{2}\right)}^{N}\sum_{j=0}^{N-K}c_{j}{\left(\frac{1-z^{-1}}{1+z^{-1}}\right)}^{j}.$$

The binomial filter in Equation (18) can be realized as a cascade of two filters:

(19) $$H_{N,K,d}\left(z\right)=A_{N}\left(z\right)B_{N,K,d}\left(z\right),$$

where

(20) $$A_{N}\left(z\right)={\left(\frac{1+z^{-1}}{2}\right)}^{N},\;B_{N,K,d}\left(z\right)=\sum_{j=0}^{N-K}c_{j}{\left(\frac{1-z^{-1}}{1+z^{-1}}\right)}^{j}.$$

**Figure 9:**
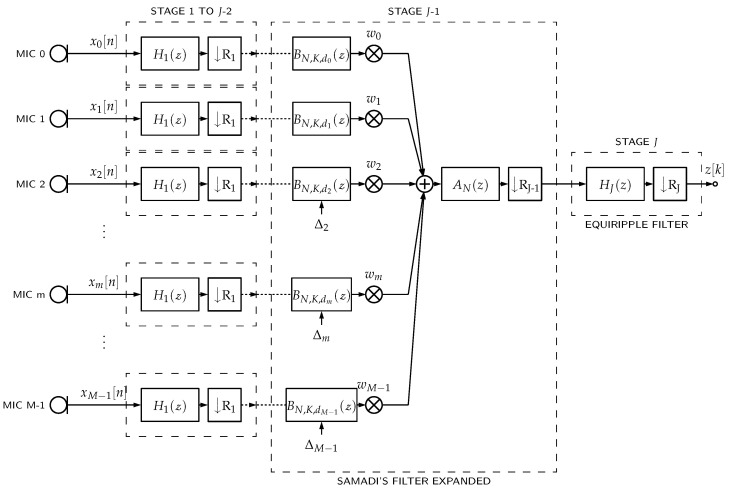
PDM-mic array DAS beamformer using delayed decimation filters.

The Samadi filter stage in a delayed decimation filter in Figure 8c can be expressed in its binomial representation in such a way that the latter part of the filter chain does not depend on Δ, as *d* is used only for the calculation of $c_{j}$. Therefore, if *M* delayed decimation filters are placed in parallel, the weightings by $w_{m}$ are placed just before the $A_{N}\left(z\right)$ filter and their outputs are added to form a beamformer. Note that the latter part after $B_{N,K,d}\left(z\right)$ can be shared between all microphone channels, as shown in Figure 9.

## 4. Proof of Concept

We now evaluate the proposed architecture. We determine the delayed decimation filter parameters for a given specification and compare the proposed architecture to state-of-the-art DAS beamformer architectures.

### 4.1. Decimation Filter Specifications

Filter specifications and array geometries change depending on the beamformer application. Therefore, to compare the efficiency between the proposed method and the straightforward DAS beamformer implementation, we use the specification shown in Table 1 as the basis of all our decimation filter designs, as it is considered enough for most PDM-mic types and speech-processing applications.

### 4.2. Beamformer Specification

The delay from the array center to the *m*th microphone ($\Delta_{m}$) in an array is constrained to

(21) $$|\Delta_{m}|\leq \Delta_{\max}\;\mathrm{for}\;m=0,1,\ldots ,M-1\;$$

such that

(22) $$\Delta_{\max}=\frac{|{\bar{x}}_{\max}-{\bar{x}}_{c}|}{c}\;,$$

where ${\bar{x}}_{\max}$ is the furthest sensor location in relation to ${\bar{x}}_{c}$ (which is the array’s center reference), *M* is the number of microphones, and *c* is the sound speed (typically 343.0 m/s).

Assume that we require a microphone array for hands-free applications that, when placed 80 cm from the voice source, would attain the same SNR as the SNR obtained by a single microphone placed 2 cm from the same source [11]. Then, by (3), the desired microphone array requires M=40 microphones.

Also, as the minimum distance between microphones should be $D_{\min}\leq c/2F_{p}$ to avoid spatial aliasing, if the frequency range is limited to $F_{p}=7.5\;\mathrm{kHz}$, then the desired microphone array will require $D_{\min}\leq 2\;cm$. Finally, as M=40, if a 5×8 microphone array is assumed, then the $\Delta_{\max}$ can be calculated using (22), with the resulting value shown in Table 2.

### 4.3. Filter Design

A delayed decimation filter was designed according to specifications listed in Table 1. The filter has a three-stage architecture ([*lthband*, *maxflat*, *equir*]) with respective decimation rates [48, 2, 2]. The *lthband* stage is an LPF whose cutoff frequency is π/L, and the impulse response is zero for every L-th sample [5]. The second stage is a *maxflat* Samadi filter, and the last is an equiripple filter [12]. As $R_{J}=R_{J-1}=2$, by (17), $d_{\max}=20.13$; the parameters *N* and *K* of the *maxflat* stage are calculated using Algorithm 1 so that the overall filter specification is kept for all |d|≤20.13.

**Figure 10:**
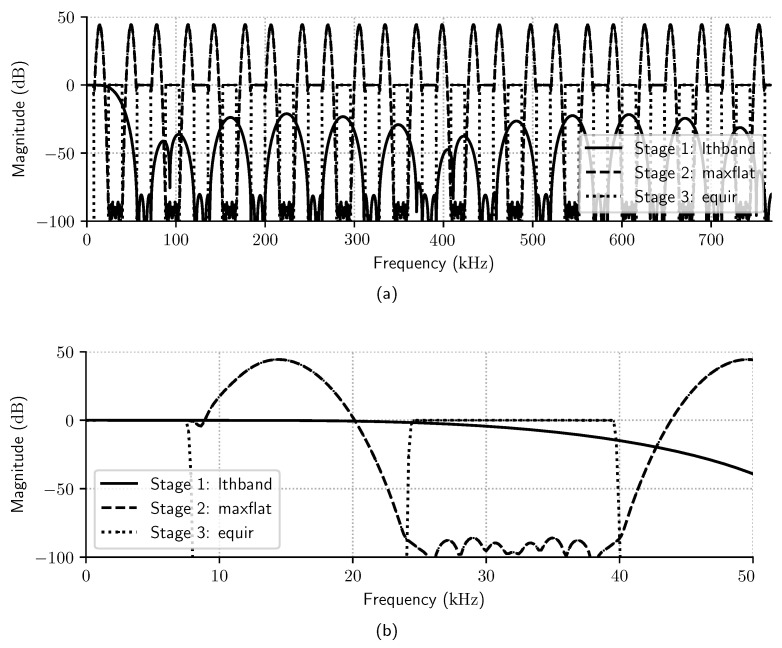
(**a**) Magnitude frequency spectrum of internal stages of the delayed decimation filter in the whole input range, and (**b**) the same frequency spectrum in the 0 kHz to 50 kHz range.

**Figure 11:**
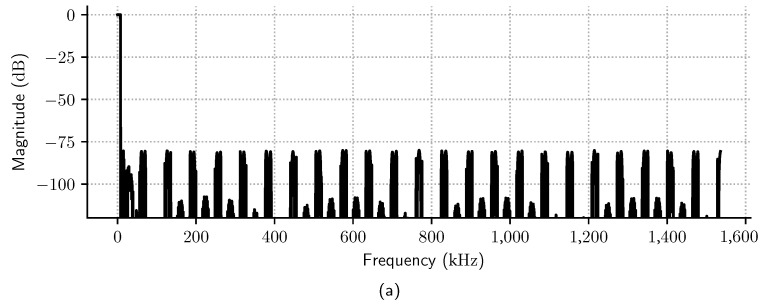

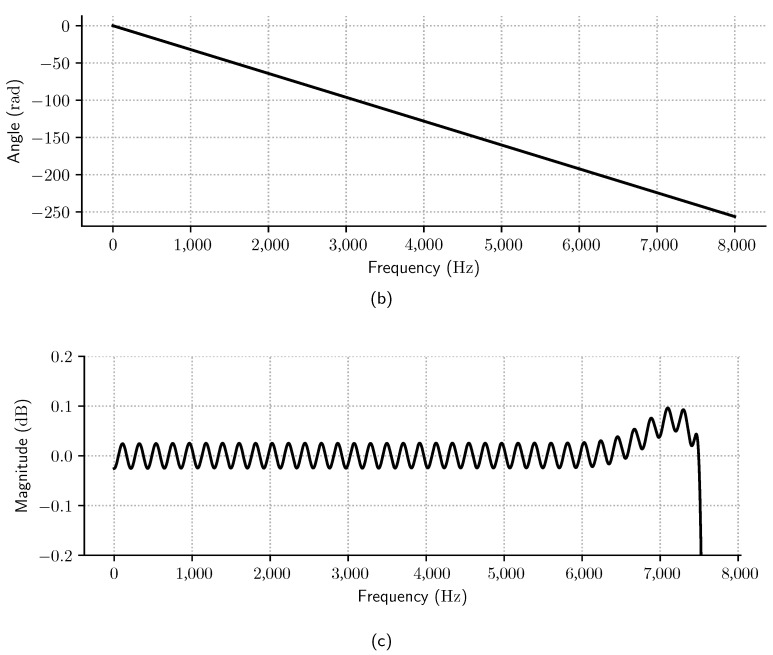
(**a**) Magnitude and (**b**) phase frequency spectrum of the delayed decimation filter. (**c**) Passband ripple frequency spectrum.

Figure 10a shows the individual frequency spectrum of each internal stage for $d_{\max}=20.13$, and Figure 10b zooms in the passband frequency region. Note that even though the *maxflat* stage has a bumpy frequency spectrum above the passband frequency ($F_{p}$), this is compensated by the last stage equiripple filter (*equir*). Figure 11a also shows that the magnitude in the overall frequency spectrum of the delayed decimation filter is inside the required passband and stopband filter specifications, while Figure 11b,c show that the filter phase and magnitude response is almost linear in the passband range.

**Figure 12:**
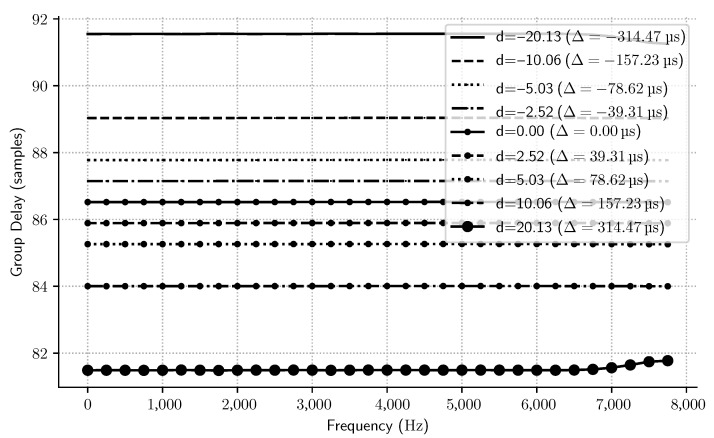
Delayed decimation filter group delay.

The advantage of using a Samadi filter is that it allows one to change its group delay by changing some coefficients, i.e., without changing the whole filter structure. Figure 12 shows the group delay of this multi-stage filter for many values of its *d* parameter. It is easy to see how the group delay is directly proportional to the *d* parameter.

Table 3 shows the resources required to implement a DAS beamformer based on this three-stage delayed decimation filter designed for array specifications listed in Table 2, and Table 4 shows the breakdown of resources required per filter stage.

## 5. Results

Results from Table 3 are compared to other state-of-the-art DAS beamformer architectures (more details in [13]) in Table 5.

The *pcm_multi* architecture is the same as shown in Figure 1 but uses a multi-stage decimator filter structure for each channel. It has more beamformer’s storage requirement and additions per second because of the parallel architecture for delaying and filtering.

The *pcm_single_memsav* architecture is also the same as shown in Figure 1 but uses a single-stage decimation filter with a memory-saving polyphase implementation [14] for each channel. This architecture has the lowest beamformer’s storage requirement because of the polyphase implementation. Still, conversely, it also has the most additions per second because more operations are performed at higher sampling rates before downsampling.

**Figure 13:**
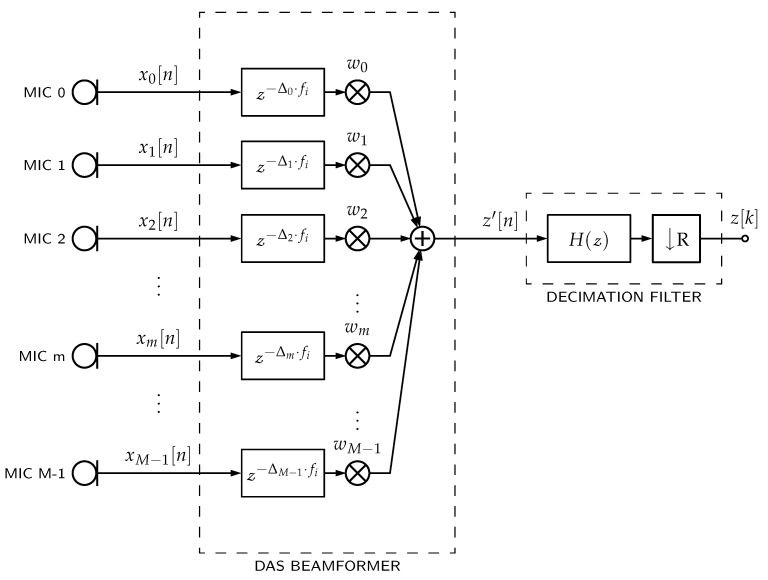
PDM microphones’ DAS beamformer at PDM domain. Each PDM-mic output $x_{m}\left[n\right]$ is delayed by a $\Delta_{m}$ factor, then all delayed signals are weighted (factor $w_{m}$) and summed together. Finally, the resulting sum is filtered and downsampled.

The *pdm_multi* architecture is the same as shown in Figure 13. Still, using a multi-stage decimator filter structure in the output is the most efficient state-of-the-art architecture because only a single decimation filter is required, and the delaying operations require only a few bits per channel.

The *pdm_single_memsav* architecture is also the same as shown in Figure 13 but using a single-stage decimation filter with a memory-saving polyphase implementation [14] in the output. It has lower beamformer’s storage requirement because of the polyphase implementation, but, conversely, it also requires more additions per second because more operations are performed at higher sampling rates before downsampling.

Table 5 shows that, for the given specification and because of the shared resources for delaying and filtering, the proposed architecture (*delayed_bf*) requires about 19% lower computational resources (additions per second) and 52% lower storage (beamformer’s storage requirement) than the most efficient state-of-the-art architecture (*pdm_multi*).

It is also observed that the proposed architecture’s storage efficiency is ranked just after the *pcm_single_memsav* architecture. However, as the *pcm_single_memsav* architecture also requires a prohibitive quantity of computational resources (about 697% more), it can be concluded that the proposed beamformer based on delayed decimation filters is the most resource-efficient beamformer architecture for the given specification.

Finally, we see that, because of the lowest computational resources requirement, in practical cases such as implementing the beamformer either in a single-core/single-adder CPU, in a Field-Programmable Gate Array (FPGA) running at 64 MHz, or in an integrated circuit (VLSI) running at 10 MHz, the proposed architecture will be, in all cases, about 19% more efficient.

## 6. Conclusions

In this study, we proposed combining the decimation filters found in PDM-mics with the delay line required in the traditional DAS beamformer. This was achieved by designing a decimation filter that includes a stage realized with the Samadi filter structure, which easily allows its group delay to be altered by the varying a single parameter.

We evaluated the proposed architecture by comparing it to other state-of-the-art DAS beamformer architectures. To facilitate the comparison, we established a set of filter specifications as a baseline for all decimation filter designs. These specifications were sufficient for various PDM-mics and speech-processing applications.

The designed filter demonstrated satisfactory performance, as exemplified in the frequency response and group delay plots. Furthermore, using a Samadi filter provided flexibility in adjusting the group delay without altering the overall filter structure.

Overall, the proposed architecture showed promising filter design and resource requirements results, providing the best trade-off between storage and computational resources. The presented specification requires 52% lower storage resources and 19% lower computational resources than the most efficient state-of-the-art architecture. The findings support the feasibility and effectiveness of the proposed approach for beamforming applications applied, but not limited, to DAS beamformers.

## Figures and Tables

**Table 1:** Decimation filter specifications.

Parameter	Value
input sampling rate ($f_{i}$)	3072.0 kHz
output sampling rate ($f_{o}$)	16.0 kHz
passband frequency ($F_{p}$)	7.5 kHz
stopband frequency ($F_{s}$)	8.0 kHz
passband ripple ($\delta_{p}$)	≤0.0116 (≤0.1 dB)
stopband ripple ($\delta_{s}$)	≤0.0001 (≤−80.0 dB)
decimation factor (*R*)	192
filter input length ($L_{\mathrm{in}}$)	1
filter output length ($L_{\mathrm{out}}$)	24
phase response	linear or almost linear

**Table 2:** Microphone array specifications.

Parameter	Value
number of microphones (*M*)	40 (5×8)
minimum distance between microphones (*D*_min_)	22.0 mm
array dimensions	110.0mm×176.0mm
maximum required delay ($\Delta_{\max}$)	314.47 μs
*m*th-filter channel gain ($w_{m}$)	1
frame length (for frequency domain implementations) (*L*_frame_)	4.0 ms

**Table 3:** Required resources to implement a beamformer using 40 shared delayed decimation filters.

	Value	Unit
beamformer’s storage requirement ($S_{\mathrm{bf}}^{z}$)	39,478	bit
beamformer’s number of multiplications per second ($S_{\mathrm{bf}}^{*}$)	6.9624 × 10${}^{8}$	MPS
beamformer’s number of additions per second ($S_{\mathrm{bf}}^{+}$)	8.81696 × 10${}^{8}$	APS
beamformer’s total number of additions per second ($S_{\mathrm{bf}}^{o}$)	2.45858 × 10${}^{9}$	APS
estimated minimum frequency in a single-core/single-adder processor (*f*_cpu_)	2458.58	MHz
estimated number of adders in an FPGA running at 64 MHz ($T_{\mathrm{FPGA}}^{+}$)	39	-
estimated number of adders in a VLSI circuit running at 10 MHz ($T_{\mathrm{lp}}^{+}$)	246	-

**Table 4:** Delayed decimation filter resource requirements breakdown. The first row corresponds to the *L*th-band filter stage, the second and third ones are to the $B_{n,k,D}\left(z\right)$ and $A_{n}\left(z\right)$ parts of the Samadi filter, respectively, and the last one to the equiripple filter.

Stage	$S_{\mathrm{bf}}^{z}$ (bit)	$S_{\mathrm{bf}}^{*}$ (MPS)	$S_{\mathrm{bf}}^{+}$ (APS)	$S_{\mathrm{bf}}^{o}$ (APS)	$f_{\mathrm{cpu}}$ (MHz)	$T_{\mathrm{FPGA}}^{+}$	$T_{\mathrm{lp}}^{+}$
*lthband*	138	15,680,000	15,616,000	15,616,000	15.62	1	2
*maxflat* — $B_{N,K,d}(z)$	552	1,536,000	6,144,000	39,936,000	39.94	1	4
*maxflat* — $A_{n}\left(z\right)$	2714	0	3,776,000	3,776,000	3.78	1	1
*equir*	9164	5,040,000	5,024,000	171,344,000	171.34	3	18

**Table 5:** Comparison of the proposed beamformer architecture based on delayed decimation filter (*delayed_bf*) and other state-of-the-art beamformer architectures implementing a DAS beamformer, as specified in Table 1 and Table 2. All percentages are related to the respective value for the *pdm_multi* architecture, the most efficient state-of-the-art architecture found for the given specification [13].

DAS Beamformer Architecture	Beamformer’s Storage Requirement ($S_{\mathrm{bf}}^{z}$) in Bit $\times 10^{3}$	Beamformer’s Total Number of Additions per Second ($S_{\mathrm{bf}}^{o}$) in APS $\times 10^{8}$	Estimated Minimum Frequency in a Single-Core/Single-Adder Processor ($f_{\mathrm{cpu}}$) in MHz	Estimated Number of Adders in an FPGA Running at 64 MHz ($T_{\mathrm{FPGA}}^{+}$)	Estimated Number of Adders in a VLSI Circuit Running at 10 MHz ($T_{\mathrm{lp}}^{+}$)
Using delayed decimation filter (*delayed_bf*)	39.5 (−52%)	24.6 (−19%)	2458.58 (−19%)	39 (−19%)	246 (−20%)
Using a multi-stage decimation filter (*pcm_multi*)	210.0 (+155%)	41.8 (+37%)	4184.94 (+37%)	66 (+37%)	419 (+37%)
Using a single-stage memory saving decimation filter (*pcm_single_memsav*)	27.4 (−67%)	243.0 (+697%)	24,303.98 (+697%)	380 (+692%)	2431 (+694%)
Using a multi-stage decimation filter at PDM domain (*pdm_multi*)	82.3 (0%)	30.5 (0%)	3050.29 (0%)	48 (0%)	306 (0%)
Using a single-stage memory saving decimation filter at PDM domain (*pdm_single_memsav*)	77.8 (−6%)	35.5 (+16%)	3553.26 (+16%)	56 (+17%)	356 (+16%)

## Abbreviations

The following abbreviations are used in this manuscript:

**Symbols**	

$B_{N,K,d}\left(z\right)$	Samadi filter binomial component.
*c*	sound speed.
$D_{\min}$	minimum distance between microphones.
$\delta_{p}^{j}$	*j*th-stage passband ripple.
$\Delta_{m}$	delay from the array center to the *m*th microphone.
$d_{\max}$	maximum allowed delay parameter.
$\Delta_{\max}$	maximum required delay.
Δ	overall filter delay.
$\delta_{p}$	passband ripple.
$\delta_{s}$	stopband ripple.
*d*	Samadi filter delay parameter.
$\delta_{s}^{j}$	*j*th-stage stopband ripple.
$f_{\mathrm{cpu}}$	estimated minimum frequency in a single-core/single-adder processor.
$f_{i}$	input sampling rate.
$f_{o}$	output sampling rate.
$F_{p}$	passband frequency.
$F_{s}$	stopband frequency.
*G*	beamformer gain.
*a*	group delay.
$H(e^{2\pi if/f_{i}})$	overall low-pass filter impulse response.
$H_{N,K,d}\left(z\right)$	Samadi filter impulse response.
H(z)	low-pass filter impulse response.
$S_{\mathrm{bf}}^{z}$	beamformer’s storage requirement.
*K*	number of zeros at z=−1 in a Samadi filter.
$L_{\mathrm{frame}}$	frame length (for frequency domain implementations).
$L_{\mathrm{in}}$	filter input length.
$L_{\mathrm{out}}$	filter output length.
*M*	number of microphones.
*N*	Samadi filter order.
*R*	decimation factor.
$T_{\mathrm{FPGA}}^{+}$	estimated number of adders in an FPGA running at 64 MHz.
$T_{\mathrm{lp}}^{+}$	estimated number of adders in a VLSI circuit running at 10 MHz.
$S_{\mathrm{bf}}^{+}$	beamformer’s number of additions per second.
$S_{\mathrm{bf}}^{*}$	beamformer’s number of multiplications per second.
$S_{\mathrm{bf}}^{o}$	beamformer’s total number of additions per second.
$U_{p}$	passband frequency range.
$U_{s}$	stopband frequency range.
$V_{p}$	passband angular frequency range.
$V_{p}^{j}$	*j*th-stage passband angular frequency range.
$V_{s}$	stopband angular frequency range.
$V_{s}^{j}$	*j*th-stage stopband angular frequency range.
$w_{m}$	*m*th-filter channel gain.
$w_{p}$	angular passband frequency.
$w_{s}$	angular stopband frequency.

**Abbreviations**	

∑ΔM	sigma–delta modulator.
ADC	analog-to-digital converter.
APS	additions per second.
DAS	delay-and-sum.
DoA	direction of arrival.
FIR	Finite Impulse Response.
FPGA	Field-Programmable Gate Array.
IIR	Infinite Impulse Response.
IoT	internet of things.
IVA	intelligent virtual assistants.
LPF	low-pass filter.
MAXFLAT	maximally flat.
MEMS	micro-electro-mechanical system.
PCM	pulse-code modulated.
PDM	pulse-density modulated.
PDM-mic	PDM microphone.
SNR	signal-to-noise ratio.
THD	total harmonic distortion.
THD+N	total harmonic distortion plus noise.
VLSI	very large-scale integration.

## References

[1] Krim H., Viberg M. (1996). Two decades of array signal processing research: The parametric approach. IEEE Signal Process. Mag..

[2] Lawes R. (2014). MEMS Cost Analysis: From Laboratory to Industry.

[3] Vardhini P.H., Makkena M.L. (2021). Design and comparative analysis of on-chip sigma delta ADC for signal processing applications. Int. J. Speech Technol..

[4] Johnson D.H., Dudgeon D.E. (1992). Array Signal Processing: Concepts and Techniques.

[5] Milic L. (2009). Multirate Filtering for Digital Signal Processing: MATLAB Applications.

[6] Metzler B. (1993). Audio Measurement Handbook.

[7] Samadi S., Nishihara A., Iwakura H. (2000). Universal maximally flat lowpass FIR systems. IEEE Trans. Signal Process..

[8] Herrmann O. (1971). On the approximation problem in nonrecursive digital filter design. IEEE Trans. Circuit Theory.

[9] Rajagpoal L., Roy S.D. (1987). Design of maximally-flat FIR filters using the Bernstein polynomial. IEEE Trans. Circuits Syst..

[10] Haddad R. (1971). A class of orthogonal nonrecursive binomial filters. IEEE Trans. Audio Electroacoust..

[11] Van Compernolle D. (2001). Future Directions in Microphone Array Processing. Microphone Arrays: Signal Processing Techniques and Applications.

[12] McClellan J., Parks T. (1973). A unified approach to the design of optimum FIR linear-phase digital filters. IEEE Trans. Circuit Theory.

[13] Carbajal Ipenza S.J. (2020). Efficient Pulse-Density Modulated Microphone Array Processing. Master’s Thesis.

[14] Fliege N. (1994). Multirate Digital Signal Processing: Multirate Systems, Filter Banks, Wavelets.

